# Glucosinolates in *Sisymbrium officinale* (L.) Scop.: Comparative Analysis in Cultivated and Wild Plants and in Vitro Assays with T2Rs Bitter Taste Receptors

**DOI:** 10.3390/molecules24244572

**Published:** 2019-12-13

**Authors:** Gigliola Borgonovo, Nathan Zimbaldi, Marta Guarise, Floriana Bedussi, Marcel Winnig, Timo Vennegeerts, Angela Bassoli

**Affiliations:** 1 Department of Food, Environmental and Nutritional Sciences-DeFENS, University of Milan, Via Celoria 2, I-20133 Milano, Italy; gigliola.borgonovo@unimi.it (G.B.); nathan.zimbaldi94@gmail.com (N.Z.); 2 Department of Agricultural and Environmental Sciences-DISAA, University of Milan, Via Celoria 2, I-20133 Milano, Italy; marta.guarise@unimi.it (M.G.); floriana.bedussi@unimi.it (F.B.); 3 IMAX Discovery GmbH, Otto-Hahn-Straße, 15, 44227 Dortmund, Germany; marcel.winnig@imaxdiscovery.com (M.W.); timo.vennegeerts.tv@axxam.com (T.V.); 4 Axxam S.p.A. Via Meucci, 3, 20091 Bresso, Italy

**Keywords:** *Sisymbrium officinale*, glucosinolates, glucoputranjivin, T2Rs bitter taste receptors

## Abstract

*Sisymbrium officinale* (L.) Scop., commonly known as “hedge mustard” or “the singer’s plant” is a wild plant common in Eurasian regions. Its cultivation is mainly dedicated to herboristic applications and it has only recently been introduced into Italy. The active botanicals in *S. officinale* are glucosinolates, generally estimated by using UV or high-performance liquid chromatography (HPLC). Using both techniques, we measured the total glucosinolates from *S. officinale* in different parts of the plant as roots, leaves, seeds, and flowers. A comparison was made for cultivated and wild samples, and for samples obtained with different pre-treatment and fresh, frozen, and dried storage conditions. Cultivated and wild plants have a comparable amount of total glucosinolates, while drying procedures can reduce the final glucosinolates content. The content in glucoputranjivin, which is the chemical marker for glucosinolates in *S. officinale*, has been determined using HPLC and a pure reference standard. Glucoputranjivin and two isothiocyanates from *S. officinale* have been submitted to in vitro assays with the platform of bitter taste receptors of the T2Rs family. The results show that glucoputranjivin is a selective agonist of receptor T2R16.

## 1. Introduction

*Sisymbrium officinale* (L.) Scop. (Brassicaceae) (SO) is a wild plant, spread mostly in the Eurasiatic Region and North Africa. SO (also commonly named “erysimum”, or “the singers’ plant”) is known for its traditional use in voice impairments and other upper respiratory trait diseases. The plant is very common in bare ground, on roadsides, dumps, and edges of fields and it is cultivated in a few countries for herbal preparations. In Italy, the cultivation of SO has been recently reported [1].

Extracts and botanical preparations from SO are commonly employed in commercial remedies for voice protection, as they show anti-inflammatory, analgesic, antitussive, expectorant [2], myorelaxant [3], and broad-spectrum antimicrobial activities [4]. The effect on alleviating vocal trait disability in a cohort of 104 patients showing various degree of vocal trait discomfort has been recently reported [5].

The chemical markers of SO are glucosinolates (GLSs) and isothiocyanates (ITCs), commonly found in all plants of the Brassicaceae family [6].

Glucosinolates, which are β-thioglucoside N-hydroxysulphate compounds, are secondary metabolites that are biosintethetically derived from amino acids. They are present in many foods and have been extensively studied for their impact on human health [7]. 

Isothiocyanates, the main breakdown products of GLSs, are volatile compounds responsible for the somatosensory sensation of pungency common in many plants of the Brassicaceae family. 

Glucosinolates and isothiocyanates represent two interesting classes of natural compounds due to their healthy properties and their role as antimicrobials and cancer preventing agents. Their properties, degradations, and applications have been extensively reviewed [8].

**Figure 1:**
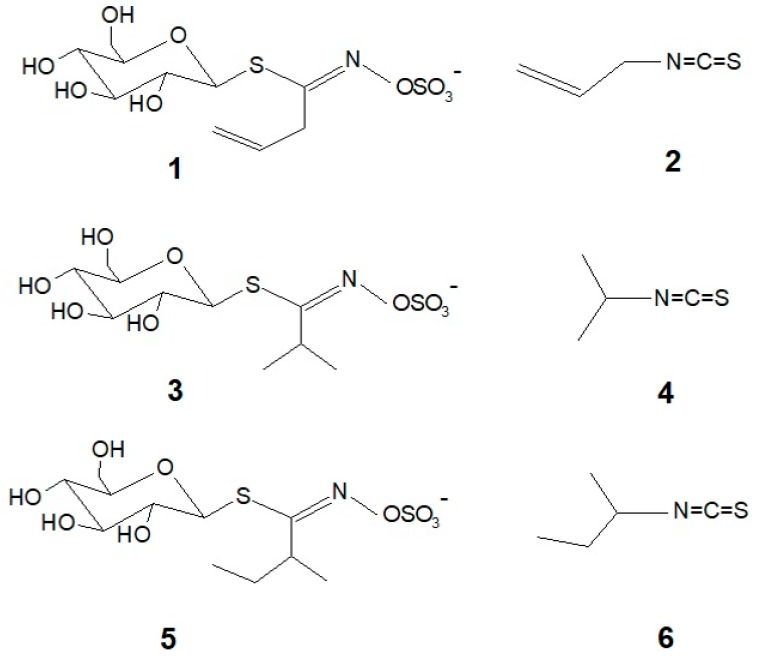
Structure of glucosinolates and the corresponding isothiocyanates in *Sisymbrium officinale*: **1** = sinigrin; **2** = allyl isothiocyanate; **3** = glucoputranjivin; **4** = isopropyl isothiocyanate; **5** = glucocochlearin; **6** = 2-butyl isothiocyanate.

One of the most studied couples of this kind is that formed by sinigrin **1** and allyl isothiocyanate **2**, (also known as mustard oil since it is commonly found in mustard (*Brassica nigra* (L.) W.D.J. Koch.) (Figure 1). Myrosinase is the specific β-glucosidase responsible for hydrolysis of GLSs to ITCs upon plant tissue disruption. For an accurate quantification of total glucosinolates the inactivation of this enzyme prior to tissue disruption is required.

Qualitative and quantitative analysis of glucosinolates in SO has previously been conducted [9] and the methodology for their isolation and dosage has been reported both in wild plant samples and in standardized preparations [10]. The main glucosinolate in SO is glucoputranjivin **3** [11], but other GLSs such as glucocochlearin **5** have also been sporadically found. The quantification of total GLSs content has been made by UV spectroscopy after derivatization with palladium salts or by high-performance liquid chromatography (HPLC); the results are expressed as sinigrin equivalents, since sinigrin is the only reference standard commercially available for GLSs.

Although the use of *S. officinale* in remedies for voice care has occurred from a long time, the mechanism of action of these phytopreparates is not known and the role of single compounds in binding specific molecular targets has not yet been studied. 

The traditional use suggests that the more efficient parts of the plant are top edges and flowers, where the content in GLSs is higher. Since isothiocyanates are formed by parent glucosinolates, both families can have a direct role in exerting a specific pharmacological activity on the vocal trait, which still needs to be investigated. 

The taste of GLSs and ITCs resulting from sensory analysis has been recently reviewed [12]. It is generally recognized that GLSs impart bitterness and ITCs impart pungency, but sensory analysis has been seldom performed on single pure compounds. The binding of such compounds to taste and somatosensory receptors can be determined using in vitro assays with cloned receptors of the T2R and TRP families. Sinigrin **1** is reported to activate receptors T2R38 and T2R16 [13] of the bitter family, whereas allyl isothiocyanate **2** is the reference agonist for TRPA1 ion channel, a sensor for pungency [14]. It has been recently reported [15] that the two main volatile ITCs from SO, isopropyl isothiocyanate **4** and 2-buthylisothiocyanate **6**, are also strong agonist in vitro of the TRPA1 somatosensory ion channel, which is involved in the somatosensory perception as well as in the nociception pathway of inflammatory pain [14,16,17]. 

On the other hand, the T2R bitter taste receptors are also known to be involved in pathologies related to the upper airways. It is known that several proteins of this family are expressed ectopically in the respiratory system in humans and other mammals. In humans they are expressed in the lungs, nose, trachea, and bronchi, and in rodents also in the vomeronasal organ. The bitter taste receptor T2R38 has been suggested to be responsible for the quorum sensing of compounds from bacterial infections [18]. Beside receptor T2R38, bitter taste receptors T2R1, T2R3, T2R4, T2R5, T2R7, T2R8, T2R9, T2R10, T2R13, T2R14, T2R16, T2R19, T2R20, T2R30, T2R31, T2R42, T2R45, T2R50, and T2R46 are also expressed in the lungs, bronchi, trachea, and nasal epithelium, where they are involved in sensing mechanisms related to defense against noxious substances from the environment or from bacterial infections [19].

The binding of GLSs from SO with the T2Rs receptors in vitro has not been previously reported. 

In this work we analyzed several samples of *S. officinale*, both wild and cultivated. We determined the total GLSs’ content as well as the content in glucoputranjivin **3**. We compared the results from cultivated plants and wild samples collected in different locations and with different soil composition. A comparison has been made among dried, freeze-dried, or frozen samples from the same plant, in order to have information about the effect of pre-treatment and storage on the final GLSs’ content. 

Finally, glucoputranjivin **3** and two isothiocyanates from SO (compounds **4** and **6**) have been assayed in vitro with the T2Rs platform of bitter taste receptors; glucoputranjivin resulted selectively active on the bitter taste receptor T2R16.

## 2. Results

### 2.1. Analysis of Total Glucosinolates in S. officinale by UV

Samples were both cultivated and wild. Plants were cultivated in the Faculty of Agriculture and Food Sciences facilities in 2017–18 [1]. Samples of wild SO were collected in four different locations, three in the city of Milan and one in a mountain region in North Italy (Val Saviore, BS). The sites have been previously identified and mapped on the project web site [20]. Wild plants were growing in different places, with some of them being difficult to reach at any time. Taking this into account, all the isolated available parts of the plant were analyzed, when possible: seeds, leaves harvested in spring and in summer, flowers, siliquae, and roots. 

For the wild samples, due to variations in exposition and soil, not all of the plants grew uniformly, therefore samples were collected as much as possible at the same stage: leaves were collected at the pre-flowering stage (leaves, summer); flowers as they appear (from May to August); siliquae and seeds at physiological maturity; roots were collected in fall, after the plant was cut. 

Samples were collected and analyzed fresh or, in most cases, they were frozen immediately after harvesting. Samples that we could not collect and immediately freeze or freeze-dry in the laboratory were preferably dried in the air.

Three commercial samples were also analyzed for comparison: two teas (entries 26 and 27) and a dry extract (entry 28).

All the analyses were performed in triplicate and the results were expressed as the average value. Samples and results of analysis are reported in Table 1.

The determination was made with a spectroscopic method [21] based on the formation of complexes of the GLSs with palladium salts. All examined samples showed a measurable GLSs content ranging from 0.41 ± 0.04 (%sinigrin eq, cultivated dry roots, entry 1) to 11.4 ± 0.26 (%sinigrin eq, fresh wild flowers, entry 20). 

As expected, the content of GLSs varies in different parts of the plant, with the flowers having the most bioactives. 

#### 2.1.1. GLSs in Different Part of the Plant

**Figure 2:**
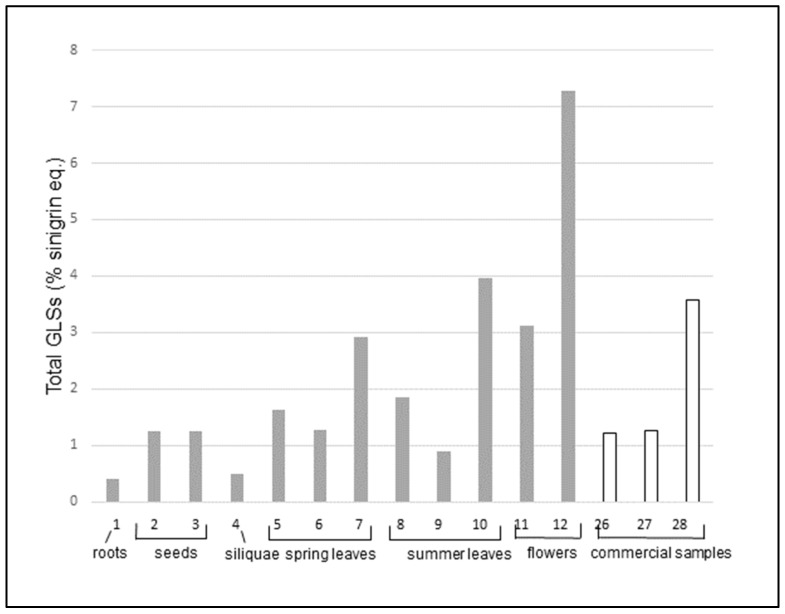
Total glucosinolates (UV) in different parts of cultivated samples of *S. officinale*: roots, seeds, siliquae, spring and summer leaves, flowers. Commercial samples (26–28) are in white.

A first comparison can be done by analyzing the data obtained for cultivated samples (entries 1-12), grown in identical media and environmental conditions. The data are shown in Figure 2.

In cultivated plants, roots and siliquae have the lowest amounts of GLS, followed by seeds. Flowers show the highest content. Dried flowers alone (entry 11) have a GLSs content more than five times higher than that of commercial teas (entries 26 and 27), which are usually made with the whole dry plant, including leaves and stems. Dried flowers alone are similar to the commercial dried extract (entry 28) that has been previously concentrated, and fresh flowers (entry 12) are two times richer than the dried extract. Leaves have an intermediate content, which do not vary substantially among spring and summer leaves. On the other hand, the content of total GLS seems to be dependent on the pre-treatment and storage methodology. In fact, the content is lower for freeze-dried leaves (entries 6 and 9), it increases when leaves are dried at room temperature (entries 5 and 8) and it has the highest value in fresh or frozen leaves (entries 7 and 10). Also, in the case of flowers, the highest concentration of total GLSs is found in fresh samples (entry 12) whereas the drying process (entry 11) decreases the GLS amount by more than fifty percent. 

#### 2.1.2. GLSs in Wild vs Cultivated Plants 

The bioactive content in secondary metabolites in plants is a highly variable parameter; soil characteristics, such as nutrients content, pH, water availability, environmental condition (climate and light exposure), and possible presence of parasites can affect it dramatically. A systematic comparison of data can be challenging due to limited access to the growing areas. During the research, we collected samples of SO in four green areas in the city of Milan, which were exposed to the same climatic conditions as the plants cultivated in the Faculty. 

**Figure 3:**
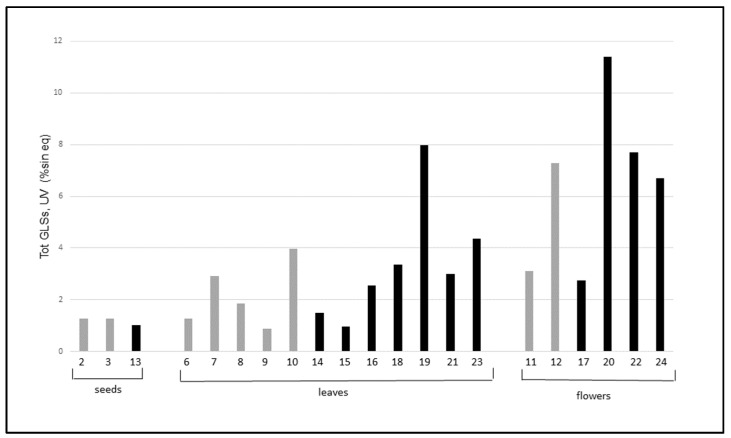
Total GLSs (UV) in samples of wild (black) and cultivated (gray) plants of *S. officinale*.

Figure 3 shows the comparison of total GLSs in the wild versus cultivated plants. 

The results show that the total GLSs content in wild and cultivated plants has a certain variability but the values for wild and cultivated samples are similar for seeds, leaves, and flowers. There are two exceptions: a sample of wild flowers (entry 20) and one of wild leaves (entry 19), which are remarkably higher than the others in the same group. Notably, both samples 19 and 20 were collected in the same area (MI2, Via Rubattino). 

To investigate the possible causes of these differences, soil samples of the three green urban areas have been collected in correspondence to each site of harvest of wild *S. officinale* (MI1, MI2 and MI3) plants and analyzed for their main chemical and physical characteristics and for their content in heavy metals [22]. The results are reported in Table 2 and Table 3.

Data reported in Table 2 show, for all three soils, a sandy- loam texture, with an equilibrate presence of silt and clay. pH values are weakly alkaline for MI 1 and M3 and neutral for M2. Total organic carbon and, consequently, organic matter content are high, in particular for MI1 and MI2 samples. MI2 is characterized for the highest CEC value and, above all, for the highest total nitrogen and available phosphorous contents. As previously reported [23,24] in studies that correlated GLSs content in plants versus soil organic matter and nutrients (i.e., nitrogen and phosphorous) in soils, effectively, and as shown in Figure 3, the samples of wild SO characterized by the highest content of GLSs were the ones grown on soil MI2, which is characterized as having a better nutritional status. 

The heavy metals content (Table 3), except for arsenic and copper, in the soil M1 exceed the limits proposed by Italian law for green areas, as expected for urban soils subjected to high levels of environmental pollution. In the soil M2, the elements that exceed the limits are nickel, zinc, and cadmium, while in the soil M3, only the nickel present a high concentration. 

Nevertheless, the concentration detected in all soils does not reaches levels that are able to cause phytotoxicity effects for plants. 

### 2.2. Comparative Analysis of Total GLSs and Glucoputranjvin by UV and HPLC Methods

For six cultivated samples (entries 1, 5, 6, 7, 8, and 12), we compared the data obtained by UV spectroscopy, as described above, with those obtained by other two analytical approaches: (1) the determination of total GLSs by RP-HPLC methodology, as described in the French Pharmacopea; (2) the determination of glucoputranjivin **3** by RP-HPLC. 

Glucoputranjivin **3** is the main GLS in *S. officinale*; its quantitative determination in all the parts of the plant has not been described previously. We performed this analysis by RP-HPLC using an authentic sample of this compound, previously obtained in our laboratory [15], as the reference standard to build a calibration curve. 

**Figure 4:**
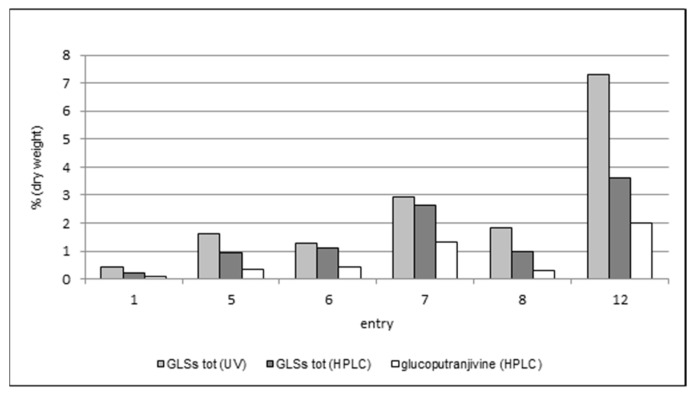
Comparison of GLSs measured by UV and high-performance liquid chromatography (HPLC) and glucoputranjivin in samples of cultivated *S. officinale*.

The results of the comparison among the three methods are summarized in Table 4 and Figure 4.

As expected, the content in glucoputranjivin **3** is lower compared to the total GLSs in each examined sample. 

For all samples, the values obtained by UV are higher than those obtained by HPLC, which could be due to the presence of interferences possibly raising the response in UV measures. This phenomenon should be overcome when using the HPLC method due to the separation of peaks. Nevertheless, this method also suffers from some uncertainty. In fact, the HPLC method described in the French Pharmacopea [10] uses sinigrin’s retention time as the reference to individuate and integrate the peaks of glucosinolates in the mixture, which are referred as “close to” or “just after” the sinigrin’s peak. The presence of sinigrin itself in *S. officinale* has been referred only sporadically, therefore the identification of this and the other peak(s) and the integration of the corresponding areas results are somewhat questionable. Using this procedure for the analysis of our samples, the results were lower than those obtained by UV, with a difference ranging from 18% (entry 6) up to 85% (entry 8).

The analysis of glucoputranjivin **3** by HPLC gave results which are compatible with the other two methodologies. The total amount of compound **3** ranges from 0.11% to 1.98%; the highest value is found in flowers (entry 12) and the lowest is in roots (entry 1). Among the leaves, sample 7 has the highest value with all three methods. 

The quantitative data obtained for compound **3** are important to evaluate its potential bioactivity in relationship to voice. To do that, we submitted glucoputranjivin **3** and other compounds to in vitro assays with the bitter taste receptors of the T2R family. 

### 2.3. In Vitro Assays of Phytocompounds from S. officinale on T2Rs Bitter Taste Receptors

**Figure 5:**
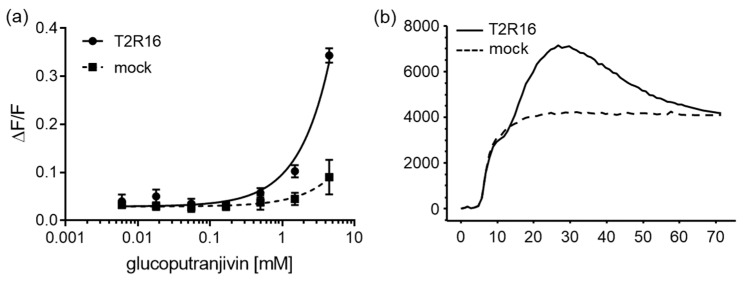
Activation of T2R16 by glucoputranjivin. (**a**) Glucoputranjivin was screened at 1 mM on all hT2Rs. Only T2R16 showed specific activation. Activation of T2R16 by glucoputranjivin was concentration dependent with an estimated EC50-value > 1 mM. (**b**) Calcium traces of hT2R16 and mock transfected cells after stimulation with 4.5 mM Glucoputranjivin. Scale: y-axis, ΔF fluorescence counts in arbitrary units; x-axis, time seconds.

In order to investigate the potential role of T2Rs in the activity of SO, we screened HEK293 PEAKrapid Gα16Gi/o44 cells which were transiently transfected with cDNAs for all 25 human T2Rs. For the primary screening, we used substance concentrations not leading to artificial activation of mock-transfected control cells that were stimulated with the same compound concentration but without receptor cDNA. From the three substances tested, the glucosinolate glucoputranjivin **3**, previously isolated from the plant, selectively activated T2R16 transfected cells (Figure 5). The two isothiocyanates **4** and **6** had inactive results.

This result is partly in agreement with those observed for other glucosinolates as sinigrin **1,** which is able to activate both T2R16 and T2R38 receptors in vitro at a concentration of 100 µM [13,25]. 

Beside sinigrin, T2R16 is also responsible for the recognition of several bitter beta-glucopiranosides [25,26]. Together with other proteins of the same family, the T2R16 receptor is expressed in the airways [19], whereas its specific role still needs to be elucidated. 

## 3. Discussion

In traditional medicine and phytotherapy, the main use of SO is to alleviate voice discomfort and mild upper airways complications. Several activities have been found in SO extracts, as antimicrobic and myorelaxant, but only few of them was clearly associated to isolated phytocompounds, as the antimutagenic activity of glucoputranjivin **3** in in vitro assays [27]. 

In a previous work we demonstrated that isopropyl isothiocyanate **4** is an effective agonist on the somatosensory ion channel TRPA1 in vitro, [15], whereas glucoputranjivin **3** is inactive on this ion channel. In the present work, we identified glucoputranjivin **3** as a selective agonist of T2R16 receptor in vitro. The result is consistent with the emerging role of the T2Rs in defense mechanisms in the airways, which have recently been proposed [28,29]. 

Comparing the activity of GLSs from mustard and those from SO, some differences can be noted. 

In mustard, sinigrin is able to activate receptors T2R16 and T2R38, the latter also being targeted by the corresponding ITC allyl isothiocyanate **2**, an effective agonist of TRPA1. In the case of erysimum, glucoputranjivin selectively activates T2R16, whereas T2R38 is not activated by compound **3** nor by its corresponding ITC 2-butyl isothiocyanate (compound **4**), which is instead active on TRPA1. 

The involvement of TRPA1 and T2Rs in the mechanism of action on voice of SO phytopreparates is still to be demonstrated by in vivo experiments and clinical data. The results obtained in in vitro assays reinforce this working hypothesis and open the possibility of further experiments in this direction, using purified compounds. For this aim, the quantitative analysis of bioactive compounds in plants is a fundamental step. Data about total GLSs and glucoputranjivin in fresh plants, both wild and cultivated, and in their derivatives has been obtained. These data are also useful to select the part of plants to be used, and the best pre-treatment and storage conditions in order to preserve the activity. 

All this information, combined with those already described in the literature, indicate that the phytopharmacology of *S. officinale* could benefit from a proper phytochemical and biological approach at the molecular level and could indicate a new approach to study the mechanism of action of these botanicals. 

## 4. Materials and Methods 

### 4.1. Chemicals

Sinigrin monohydrate (potassium salt), methanol, acetonitrile, ammonium acetate, sodium tetrachloropalladate, and hydrochloric acid were obtained from Sigma-Aldrich (Milan, Italy). 

HPLC analysis were performed with a liquid Chromatograph Dynamax SD200 (VARIAN^®^-Rainin, Woburn, US), equipped with a binary pump with a Rheodyne injector and a UV-VIS detector managed by the Galaxy Chemstation. A reversed phase column C18 Lichrosphere (250 mm length, 4.6 mm ID, 5 μm, Phenomenex^®^) was used. The samples were filtered with 0.45 μm nylon filters. The conditions used are the following: flow 0.7 mL/min, λ 227 nm; solution A: ammonium acetate 0.01 M; solution B: acetonitrile; gradient elution initially 100% of A for 10 min, in 5 min 95% of A for 10 min, at 45 min 30% of A. 

UV spectra were registered on a Perkin–Elmer Lamba 2 UV/VIS spectrophotometer, with a 1 cm cuvette and an integration time of 25 s. 

A Heto Drywinner instrument (A. De Mori) was used for lyophilization of samples. For hot air drying we used a home dryer (Bomann instrument). A Binder oven was used to dry the samples.

### 4.2. Plant Material 

*Sisymbrium officinale* (L.) Scop. was cultivated in a greenhouse at Faculty of Food and Agricultural Sciences at the University of Milan [1] and plants were harvested during 2017 and 2018. 

Samples of wild SO were collected in four different locations, three in the city of Milan and one in a mountain region in North Italy (Val Saviore, BS). The sites have been previously identified and delocalized on the project web site [20]. 

Wild plants have been identified by standard procedures by Simon Pierce and Luca Giupponi, University of Milan. 

Leaves, flowers, roots, siliquae and seeds were collected and analyzed. In particular, the leaves analyzed were fresh, frozen, dried at room temperature, and freeze-dried. 

Two commercial samples were analyzed: herbal tea (entry 26) from “L’ape regina” (Milan, Italy); herbal tea (entry 27) from Praglia Abbey, Teolo (Padova, Italy). The dry extract (entry 28) was kindly supplied by EPO, S.r.l. (Milano), batch number 1600363, code number 3113305. 

### 4.3. Sample Preparation

Each sample was obtained by sampling three different plants by taking leaves or flowers at different plant heights. 

The plant material was dried and stored at room temperature under light-protected and humidity-proof conditions or frozen and stored in refrigerator. The material was ground before chemical analysis. Leaves before freeze-drying were frozen at −20 °C. The aliquot dried at room temperature was kept in shade until it was completely dried. 

For the determination of dry mass, the plant material (1.00 g) was kept in an oven at 105 °C for several hours until the difference in weight in two consecutive weightings was less than 1 mg. The average weight loss was 81%.

Leaves, flowers, roots, and seeds were extracted separately. Portions of 1.00 g of each were extracted with methanol 55% according to the French Pharmacopeia method [10]. The extracts were submitted for glucosinolates quantification by spectrophotometry and by HPLC. Before HPLC analysis, each crude extract was passed into a small RP 18 column in MeOH and filtered with a 0.45 nylon filter.

### 4.4. Spectrophotometry

Spectrophotometric estimation of total glucosinolates content was done in accordance with Mawlong [21] with 100 μL of extract. Briefly 0.3 mL double distilled water and 3 mL of 2 mM sodium tetrachloropalladate (58.8 mg Sodium tetrachloropalladate + 170 μL concentrated HCl + 100 mL double distilled water) was added to it. After incubation at room temperature for 1 h, absorbance was measured at 425 nm. The complex forming by GLS and palladate reagent shift from light brown to dark depending on the glucosinolates concentration. The calibration curve was built with six standard solutions of sinigrin with concentrations ranging from 0.50 to 2.00 mM. The total glucosinolates (t-GSL) content were expressed as % of sinigrin equivalents (%sinigrin eq) on dried material (DW).

### 4.5. HPLC Quantification

For total GLS quantification the calibration curve was determined with six standard solutions of sinigrin with concentrations ranging from 0.25 to 1.20 mM in methanol. The total glucosinolates content was expressed as % of sinigrin equivalents on dried material (DW) and was calculated by summing the areas of glucoputranjivin and the area of peaks with the retention time near the sinigrin and an area of peak with the retention time higher with respect to sinigrin, as suggested by French Pharmacopeia. In our analytical condition, sinigrin has a Tr of 4.66 min while glucoputranjivin has a Tr of 5.18 min. We considered the peaks that elute between 4.30 and 5.30 min as an integration interval. Pure glucoputranjivin, isolated and purified by *S. officinale,* as was recently reported by us [14], was used as a standard to quantify the content in different plant extracts. The calibration curve was determined by six samples of glucoputranjivin with concentrations ranking from 8.0 to 520 µg/mL of methanol, while the linearity of the calibration curve was adequate (R > 0.993). Glucoputranjivin content was reported as mg/g DW and as a percentage of DW.

### 4.6. T2R Constructs

The coding regions of hT2R genes were either synthesized or amplified from HEK293 cell genomic DNA. All T2R sequences were tagged with an amino-terminal rSST3-tag coding for the 45 amino-terminal amino acid residues of rat somatostatin receptor subtype 3 to improve plasma membrane translocation and were cloned into a pcDNA5 expression vector.

### 4.7. Functional Expression of T2Rs

For functional analysis HEK293 PEAKrapid Gα16i/o44 cells were transfected with Lipofectamine2000 (Invitrogen) according to the manufacturer’s instructions and seeded into poly-D-lysine coated 384-well plates. After 24 h, cells were loaded with fluorescent dye (Cal520-AM, AAT Bioquest), 2 μM in Tyrode’s buffer (130 mM NaCl, 5 mM KCl, 1 mM MgCl_2_, 5 mM NaHCO_3_, 2 mM CaCl_2_, and 20 mM HEPES in water at pH 7.4) and incubated for at least 3 h at room temperature. To remove residual dye, cells were washed once with Tyrode’s buffer. Test compounds were diluted in Tyrode’s buffer and maximal concentrations of test compounds were individually adjusted to avoid the induction of unspecific signals in mock-transfected cells. Calcium responses of transfected cells upon test compound application were measured using a Fluorometric Imaging PlateReader, FLIPR Tetra (Molecular Devices). Data were collected from at least two independent experiments carried out in quadruplicates. For the calculation of dose-response curves, signals of four wells receiving the same concentration of test substances were averaged. Signals were normalized to background fluorescence. For the calculation of EC50 values, plots of amplitude versus concentrations were prepared in PRISM and fitted by using nonlinear regression.

## Figures and Tables

**Table 1:** Total glucosinolates (GLSs) content determined by UV in wild and cultivated plants and in commercial samples of *S. officinale*.

Entry	Type ^1^	Part of the Plant ^2^	Pre-Treatment ^3^	GLSs ± sd (UV, % sin eq)
1	cultivated	roots	dry	0.41 ± 0.04
2	“	seeds	-	1.26 ± 0.03
3	“	seeds	defatted	1.26 ± 0.01
4	“	siliquae	dry	0.50 ± 0.02
5	“	leaves, spring	dry	1.63 ± 0.06
6	“	leaves, spring	freeze-dry	1.27 ± 0.20
7	“	leaves, spring	freeze	2.92 ± 0.17
8	“	leaves, summer	dry	1.85 ± 0.11
9	“	leaves, summer	freeze-dry	0.89 ± 0.09
10	“	leaves, summer	fresh	3.96 ± 0.07
11	“	flowers	dry	3.11 ± 0.08
12	“	flowers	fresh	7.29 ± 0.16
13	wild, MI1	seeds	-	1.03 ± 0.02
14	“	leaves, summer	dry	1.50 ± 0.06
15	“	leaves, summer	freeze-dry	0.96 ± 0.05
16	“	leaves, summer	fresh	2.54 ± 0.04
17	“	flowers	fresh	2.75 ± 0.19
18	wild, MI2	leaves, summer	dry	3.36 ± 0.18
19	“	leaves, summer	freeze	7.97 ± 0.25
20	“	flowers	fresh	11.4 ± 0.26
21	wild, MI3	leaves, summer	freeze	3.00 ± 0.88
22	“	flowers	freeze	7.69 ± 1.81
23	wild, MI4	leaves, summer	freeze	4.37 ± 0.40
24	“	flowers	freeze	6.69 ± 0.28
25	wild, VS	flowered aerial parts	dry	1.76 ± 0.11
26	tea, 1	flowered aerial parts	dry	1.22 ± 0.10
27	tea, 2	flowered aerial parts	dry	1.26 ± 0.03
28	dry extract	extract	powder	3.58 ± 0.05

^1^ Type of samples: cultivated plants were grown in the Faculty farms; wild samples: Wild MI1: Milano, Via Colombo; Wild MI2: Milano, Via Rubattino; Wild MI3: Milano, Via Bisceglie; Wild MI4: Milano, Via Ippodromo; Wild VS: Val Saviore valley (BS). ^2^ Spring leaves were collected between February and March; summer leaves were collected just before the flowering, between April and June. ^3^ Seeds were collected and stored at room temperature; details are described in the experimental part.

**Table 2:** Analysis of soil in the growing areas of wild SO.

Site ^1^	Silt	Clay	Sand	Classification (USDA)	pH	TOC ^2^	Tot N	OM ^2^	C/N ^2^	CEC ^2^	P_2_O_5_
	% dry soil					g·kg^-1^				cmol^+^·kg^−1^	mg·kg^−1^
MI1	6	19	75	sandy loam	7.40	49.8	1.70	85.6	29.3	11.3	105
MI2	6	20	74	sandy loam	7.01	49.0	3.43	84.2	14.3	22.6	267
MI3	6	21	72	sandy loam	7.52	28.8	1.64	49.5	17.5	14.3	93

^1^ MI1: Milano, Via Colombo; MI2: Milano, Via Rubattino; MI3: Milano, Via Bisceglie. ^2^ TOC = Total Carbon Content; OM = Organic Matter; C/N = Carbon to Nitrogen content; CEC = Cation Exchange Capacity; P_2_O_5_ = phosphorous content as phosphorous oxide.

**Table 3:** Soils heavy metals content.

Entry	Cr	Ni	Cu	Zn	As	Cd	Pb
	(mg·kg^−1^) ^1^						
MI1	205	357	104	562	13.3	10.7	558
MI2	147	364	112	344	4.62	3.08	95.1
MI3	129	390	61.4	126	4.01	1.93	50.1
Limits Italian Law 471/1999	150	120	120	150	20	2	100

^1^ data expressed on the basis of dry soil and determined by ICP-MS after acid digestion in microwave by HNO_3_.

**Table 4:** Comparison among total GLSs content determined by UV and by HPLC and glucoputranjivin content by HPLC in six samples of cultivated *Sisymbrium officinale*.

Entry	Tot GLSs ± sd (UV, % sin eq) ^1^	Tot GLSs ± sd (HPLC, % sin eq) ^1^	Cpd 3 ^2^ ± sd (HPLC, %) ^3^
1	0.41 ± 0.04	0.23 ± 0.01	0.11 ± 0.02
5	1.63 ± 0.06	0.94 ± 0.08	0.34 ± 0.02
6	1.27 ± 0.20	1.09 ± 0.20	0.41 ± 0.01
7	2.92 ± 0.17	2.63 ± 0.01	1.32 ± 0.01
8	1.85 ± 0.11	1.00 ± 0.04	0.30 ± 0.02
12	7.29 ± 0.16	3.63 ± 0.44	1.98 ± 0.04

^1^ data are expressed in % of sinigrin equivalents; ^2^ cpd 3 = compound **3**, glucoputranjivin; ^3^ data are expressed in % dry weight.
